# Influence of autophagy on acute kidney injury in a murine cecal ligation and puncture sepsis model

**DOI:** 10.1038/s41598-018-19350-w

**Published:** 2018-01-18

**Authors:** Satoshi Sunahara, Eizo Watanabe, Masahiko Hatano, Paul E. Swanson, Takehiko Oami, Lisa Fujimura, Youichi Teratake, Takashi Shimazui, Chiwei Lee, Shigeto Oda

**Affiliations:** 1 Department of Emergency and Critical Care Medicine, Graduate School of Medicine, Chiba University, Chiba, Japan; 2 Biomedical Research Center, Chiba University, Chiba, Japan; 3 Department of Biomedical Science, Chiba University Graduate School of Medicine, Chiba, Japan; 4 Department of Pathology, University of Washington School of Medicine, Seattle, USA; 5 Department of Nephrology, Chiba University Graduate School of Medicine, Chiba, Japan

## Abstract

The role of autophagy in the maintenance of renal homeostasis during sepsis is not well understood. We therefore aimed to determine the influence of autophagy on kidney during sepsis using a murine sepsis model, i.e. cecal ligation and puncture (CLP). In CLP treated animals, the number of autolysosomes observed by electron microscopy increased over time. The number of autophagosomes in CLP animals decreased relative sham operated controls at 24 hrs after CLP, indicating that autophagy flux is already diminishing by that time. Moreover, CLP induced an increase in LC3-II/LC3-I ratio at 6–8 hrs, demonstrated in western blots, as well as an increase in GFP-LC3 dots at 6–8 hrs and 24 hrs, using immunofluorescence and anti-LC3 and LAMP1 antibodies on tissue sections from GFP-LC3 transgenic mice. LC3-II/LC3-I ratio and the number of co-localized GFP-LC3 dots and LAMP1 signals (GFP LC3 + LAMP1 dots) in CLP mice at 24 hrs were significantly reduced compared with data obtained at 6–8 hrs. Notably, acceleration of autophagy by rapamycin resulted in improvement of renal function that was associated with improvement in the histologic severity of tubular epithelial injury in CLP treated animals. Autophagy in the kidney was significantly slowed in the kidney during the acute phase of sepsis; nonetheless, autophagy in kidney appears to play a protective role against sepsis.

## Introduction

Sepsis continues to be associated with a high mortality rate, despite significant advances in intensive care and an improved understanding of the pathophysiological processes underlying disease development^1^. Multiple organ failure in sepsis is a consequence of cellular dysfunction in the affected organs^2,3^, and studies of the underlying mechanisms leading to cellular dysfunction have shifted from a focus on necrosis to processes associated with apoptosis (type I programmed cell death)^4^. However, the literature suggests that while apoptosis of immune cells appears to be involved in immune paralysis in subacute sepsis^5,6^, apoptotic cell death is not prominent in either hepatic or renal epithelial cells in patients with sepsis^7^. Autophagy (type II programmed cell death) is therefore speculated to play an active role in both kidney and liver in early sepsis. In the present study, we focused on the role of autophagy in kidney epithelial injury and renal function during sepsis.

Autophagy (“auto-digestion”) degrades damaged or senescent organelles and proteins^8–10^. The major role of autophagy is the starvation response to maintain nutritional status, which is essential for the survival of living organisms^11^. The degraded macromolecules are returned to the cytoplasm for energy metabolism or anabolic reactions^12,13^. Importantly, autophagy is not just a mechanism to mitigate starvation; it has myriad essential influences on cell function and survival, including the degradation of unnecessary organelles, tumor suppression^14,15^, and the inactivation of pathogenic organisms. However, excessive autophagy can cause unwanted and deleterious cell death^8^.

In sepsis, autophagic processes are involved in the removal of damaged mitochondria^16^ and increased numbers of autophagic structures are present in the hepatocytes of patients with severe sepsis^17^. The potential roles for autophagy in sepsis are suggested by the contexts in which departures from baseline autophagy, through signaling mechanisms or cross-talk with other pathways of cell injury and death^18^, may occur. A notable example of this phenomenon is the physiologic response to severe infection and the capacity of autophagy to eliminate pathogenic organisms^19,20^. Methods for analyzing autophagy include the measurement of autophagy-related proteins, imaging after fluorescent immunostaining, and imaging by electron microscopy^21^. We previously reported that autophagy stagnated over time in the liver of septic mice and that inhibiting autophagy in septic mice worsened liver function and survival^22^. To better define this effect we recently generated liver-specific autophagy-deficient (Alb-Cre^ERT2^/Atg5^f/f^) mice and confirmed the protective role of autophagy in sepsis pathophysiology through regulation of apoptosis^23^.

To date, studies have shown that autophagy may play a protective role in organs such as the heart^24,25^, lungs^26^, liver^22,27^ and immune-competent cells^28^ during sepsis. However, there are only few reports on the relationship between septic acute kidney injury (AKI) and autophagy^29^. We thus investigated the roles of autophagy, including a dynamic morphologic classification of autophagic structures and their relative changes over time, in the pathogenesis of septic AKI using the cecal ligation and puncture (CLP) mouse model of sepsis.

## Methods

### Animals

Male C57BL/6 N (6- to 8-week-old) mice and green fluorescent protein (GFP)- microtubule-associated protein light chain 3 (LC3) transgenic mice (C57BL/6 background; 6- to 8-week-old) were acclimated to a 12-hr day/night cycle under specific pathogen-free conditions with food at least 1 week before experiments. All experimental procedures were approved by the Institutional Animal Care and Use Committees of Chiba University (ref.# 29–287) and were in compliance with the National Institute of Health guidelines.

### Cecal Ligation and Puncture (CLP) model

Sepsis was induced by CLP as described previously^17^. Briefly, mice were anesthetized with isoflurane and after laparotomy, the cecum was ligated with a 3–0 silk tie and punctured with a 23-gauge needle at two sites, followed by expression of a small amount of fecal material into the peritoneal cavity. After surgery, 1 ml of 0.9% saline was injected subcutaneously. Sham-operated mice were treated with the same procedure, but without cecum ligation and puncture. No antibiotics or analgesics were used, and mice were food-deprived but had free access to water postoperatively. In selected animals, rapamycin (10 mg/kg) was injected intra-peritoneally 1 hr after the operation, a dose drawn from recent studies that have established the efficacy of rapamycin in similar investigative contexts^24,30^. Mice were sacrificed at indicated time points after treatment and tissue samples were taken for analysis. Mice were sacrificed when they were moribund.

### Western blot analysis

Total proteins were prepared from mouse kidneys. Each tissue was lysed in 2 × SDS sampling buffer (1.245 M Tris-HCl at pH 6.8 containing 10% glycerol, 4.6% sodium dodecyl sulfate, 10% 2-mercaptoethanol, and 0.04% bromophenol blue). Extracts were homogenized on ice and boiled for 5 minutes; these were then centrifuged at 10,000 × g for 10 minutes at room temperature, and the supernatants were obtained as total protein. Equal amounts of protein were separated by SDS polyacrylamide gel electrophoresis and transferred to polyvinylidene difluoride membrane. The membranes were subsequently incubated with 5% nonfat dry milk in Tris-buffered saline (TBS) containing 0.1% Tween-20 (TBS-T) for 1 hr at room temperature. Antibodies were added and incubated overnight at 4 °C in TBS-T. The following primary antibodies were used: rabbit polyclonal GAPDH (Abcam, Cambridge, UK, 1:1000), rabbit polyclonal anti-LC3B (Sigma-Aldrich, St. Louis, 1:1000), rabbit polyclonal anti-p62 (DakoCytomation, Glostrup, Denmark, 1:500), rabbit monoclonal Rubicon (Cell Signaling Technology, Danvers US, 1:1000). Membranes were washed 3 times in TBS-T and subsequently incubated with peroxidase conjugated secondary antibodies (goat anti-rabbit IgG: Jackson Immuno Research, West Grove, PA, 1:2000; goat anti-mouse IgG: Jackson Immuno Research, 1:2000). Blots were washed 3 times with TBS-T and once with TBS, and the signal was then detected using enhanced chemiluminescence (ECL-Plus) reagent (GE Healthcare, Piscataway, NJ, USA). Band images were scanned and densitometric analysis was performed using NIH Image software (Bethesda, MD, USA). Quantification data, evaluated by band-intensity of LC3-I and -II, were normalized to that of GAPDH.

### Immunofluorescent microscopy

Mice were transcardially perfused with 4% paraformaldehyde (PFA) in phosphate buffer. Kidneys were removed and were further fixed with 4% PFA at 4 °C overnight. The kidneys were then placed in 15% sucrose in PBS at 4 °C for 4 hrs; this was then exchanged for 30% sucrose in PBS, and incubation continued at 4 °C overnight. The kidneys were frozen in optimum cutting temperature (OCT) compound and sectioned serially into 7-μm-thick sections using a cryostat. Samples were kept frozen at −80 °C until used. For immunofluorescence, sections were stained using rabbit polyclonal anti-lysosome-associated membrane protein type 1 (LAMP1) (Abcam, Cambridge, UK; 1:1000). Cy3-conjugated goat anti-rabbit immunoglobulin G (IgG) (H + L) was used as a secondary antibody (Jackson Immuno Research; 1:1000 dilution). All fluorescence images were digitally acquired at 400× magnification with KEYENCE Fluorescence Microscope BZ-X700 (KEYENCE Co., Osaka, Japan).

### Electron microscopic analysis

Samples were fixed with 2% PFA, and 2% glutaraldehyde in 0.1 M phosphate buffer, pH 7.4, at 4 °C overnight. After fixation and dehydration, 70 nm sections were prepared with a diamond blade, using an ultramicrotome (ULTRACUT UCT; Leica) and mounted on metal grids. These were stained with 2% uranyl acetate and secondarily stained with lead solution and examined with a transmission electron microscope (JEM-1200EX; JEOL Ltd.). Specimens were examined as previously described^22^. Briefly, a minimum of 8–10 random fields (to minimize unintended sampling bias) were examined at 2,500× magnification for evidence of autophagy or cell injury/death, and the number of autophagosomes and autolysosomes in each 2500× image was counted. The median ± interquartile per 50 images from each mouse was calculated and the data from different groups were compared (CLP (n = 3) versus Sham (n = 3)) at the time course of 6–8hrs and 24hrs after each surgery.

In the present study, autophagosomes were strictly defined as double membrane structures that enclosed cytoplasm with damaged organelles in various stages of degradation; double membrane structures enclosing only materials that resembled background cytoplasm were not counted. Autolysosomes were defined as single membrane vesicles with cytoplasmic or organellar debris in various stages of degradation (usually less obviously the remains of specific organelles compared with autophagosomal contents). Lysosomes with amorphous electron dense material (heterolysosomes) were not counted. The 2,500× survey images used in this analysis represent approximately 3,000 square microns of tissue, each containing cuboidal cells of the proximal convoluted tubule. The latter exhibit the following features: a brush border, a junctional complex, plicae and folds located on the lateral surface of the cells, extensive integration of basal processes of adjacent cells, and basal striations which consist of elongate mitochondria concentrated in the basal processes and oriented vertically to the basal surface^31^. Only the proximal tubular epithelial cells were evaluated.

### Analysis of renal function parameters

Blood samples were obtained from heart chamber of mice (n = 6). Serum concentration of Cystatin C was measured using an enzyme immunoassay kit (R&D, Minneapolis, MN). Blood urea nitrogen (BUN) and serum creatinine were measured by SRL company.

### Histological analysis

Kidney tissue specimens were obtained and sections of formalin-fixed paraffin-embedded kidney samples were stained with hematoxylin-eosin and examined to assess the degree of kidney injury. Renal tubular cells of the cortex were examined by two experts in a blind manner and were scored according to the percentage of damaged tubules per all tubules: 1, <25% damage; 2, 25 to 50% damage; 3, 50 to 75% damage, 4, 75 to 90% damage; and 5, >90% damage. Renal tubular epithelial cells in which cell shedding and chromatin condensation were observed were defined as injured renal tubules^32^. Renal tubules in 5 high power fields (HPFs), including 50–100 tubules per sample, were evaluated and scored.

### Statistical analysis

All data were analyzed for statistical significance using the unpaired t test or Mann-Whitney test. All data were expressed as the median ± interquartile range using the statistical software program PRISM (GraphPad Software, San Diego, CA, USA).

## Results

### Evaluation of autophagy flux in the kidney of the septic model mice

**Figure 1:**
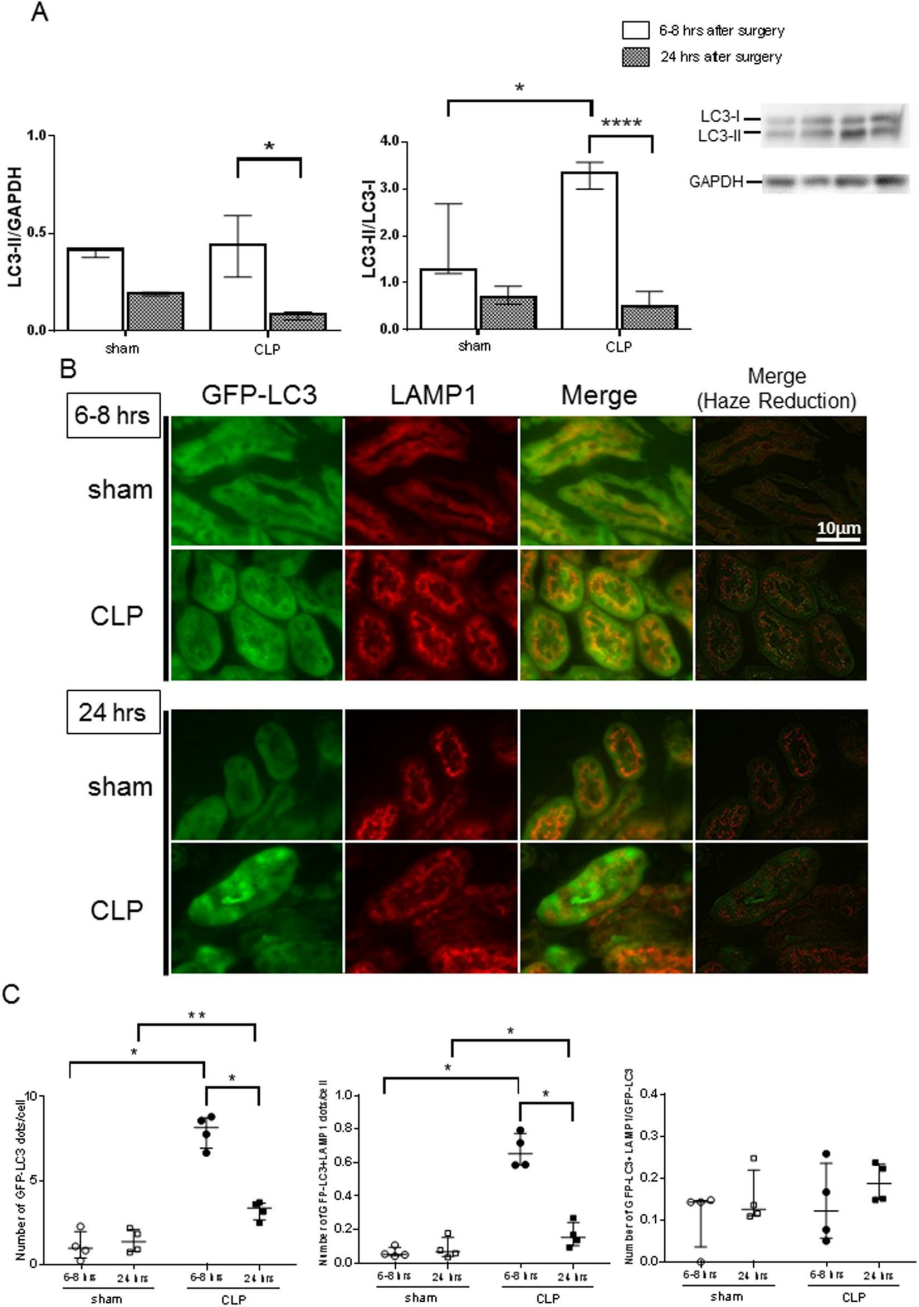
Cecal ligation and puncture (CLP) induces autophagy in the kidney of CLP model mice. (**A**) Western blotting analysis of microtubule-associated protein light chain 3 (LC3) in the kidney. Sepsis was induced by CLP. Kidney samples were prepared from sham-operated and CLP mice at each indicated time point after surgery. The left graph is the ratio between the levels of LC3-II and GAPDH, and the right graph is the ratio between the levels of LC3-II and LC3-I at each time point. All data are expressed as median ± interquartile range. Data were analyzed for statistical significance using the unpaired t test (*P < 0.05, n = 4 in each group). Whole image of the blot is shown in supplementary information. (**B**) Co-localization of green fluorescent protein (GFP)-Microtubule-associated protein light chain 3 (LC3) dots with lysosome associated membrane protein type 1 (LAMP1) in the kidney after cecal ligation and puncture (CLP) or sham surgery. Confocal images of kidney samples obtained from GFP-LC3 transgenic mice. LAMP1 was stained using Cy3-conjugated IgG secondary antibodies. Merged images demonstrate co-localization of GFP-LC3 dots and LAMP1. The number of GFP-LC3 and GFP-LC3 + LAMP1 dots per cellular confocal image was quantified at 6–8 and 24 hrs after CLP. (**C**) We counted the number of dots within entire renal tubular cells and dots within glomerular cells were not counted. The ratio of GFP-LC3 and GFP-LC3 + LAMP1 dots was calculated. All data are expressed as median ± interquartile range. Data were analyzed for statistical significance using the Mann-Whitney test. (*P < 0.05, **P < 0.01; n = 50 cells /animal; n = 4 animals).

In order to examine the induction and flux of autophagy in the kidney in a murine septic model, we examined autophagy kinetics in kidney samples by two means. First, CLP induced an increase in microtubule-associated protein light chain 3 (LC3)-II/LC3-I ratio at 6–8 hrs; however, both the LC3-II/LC3-I ratio and the LC3-II/GAPDH ratio were reduced significantly by 24 hrs after the surgical insult in C57BL/6 N mice (Fig. 1A). Second, autophagosome formation in the kidney was examined using GFP-LC3 transgenic mice. In these mice, autophagosomes are visualized as cytoplasmic GFP-LC3 dots by immunofluorescent microscopy. CLP induced an increase in GFP-LC3 dots at 6–8 hrs and 24hrs (Fig. 1B). Interestingly, however, the number of GFP-LC3 dots in CLP mice at 24 hrs compared with CLP mice at 6–8 hrs was reduced significantly (P < 0.05, Fig. 1C).

In order to further analyze the process of autophagy, fusion of autophagosomes with lysosomes was examined by immunofluorescence. Co-localization of GFP-LC3 dots and immunofluorescent signals for the lysosomal marker LAMP1 (GFP LC3 + LAMP1 dots) was evaluated in the kidney after CLP. The number of GFP-LC3 + LAMP1 dots at 6–8 hrs and 24 hrs in CLP was significantly increased when compared to sham operated controls, although (in a manner similar to GFP-LC3 dots) the number of GFP-LC3 + LAMP1 dots in CLP mice at 24 hrs compared to 6–8 hrs was reduced significantly (Fig. 1C). This indicates that formation of autophagic structures accelerates early in CLP, but attenuates over time.

### Morphological evaluation with transmission electron microscopy in septic kidney

**Figure 2:**
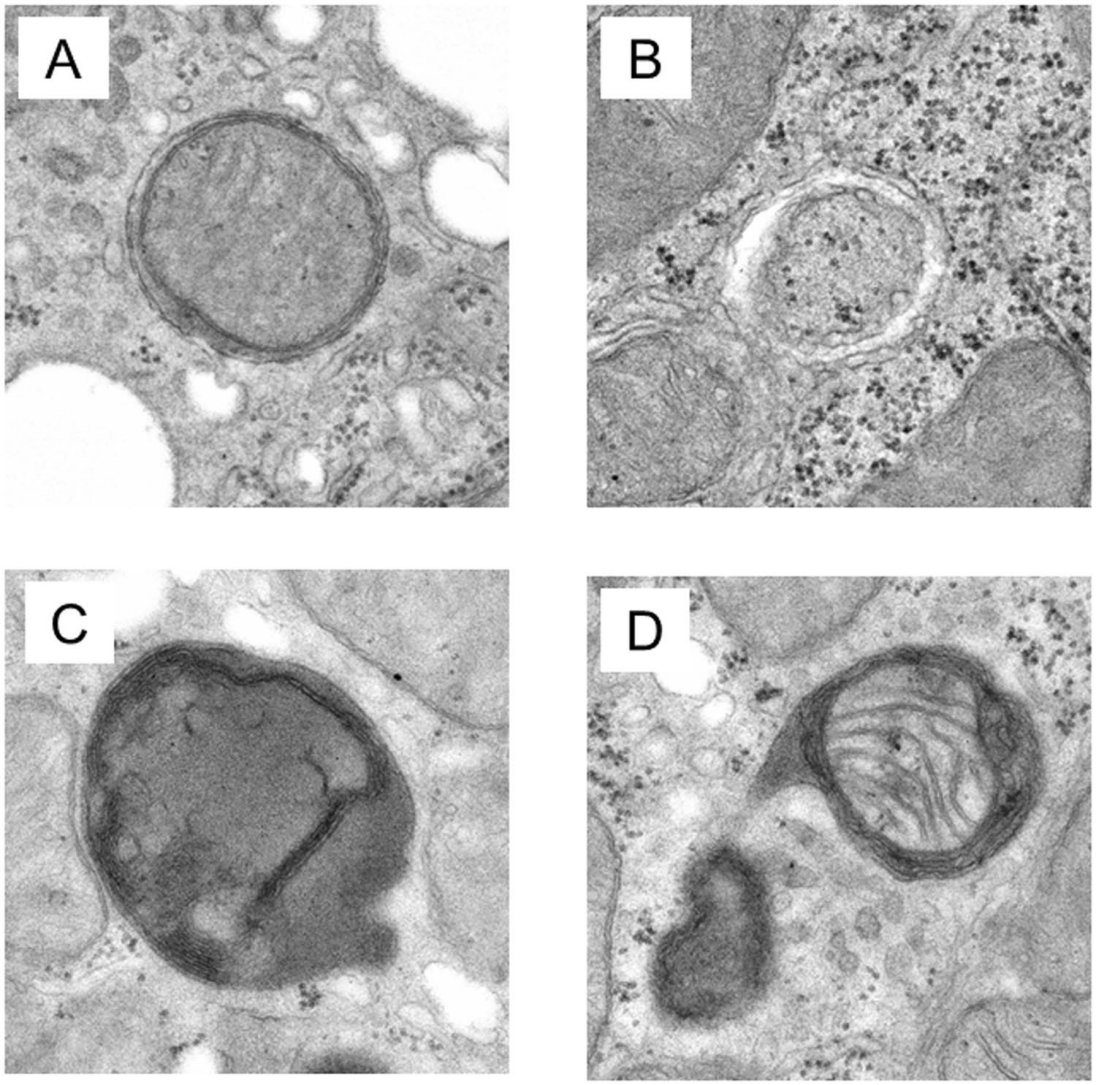
Classification of Autophagosome/Autolysosome. (**A**) An autophagosome with clear double membrane structures. (**B**) An autophagosome being engulfed process or just recently engulfed by a sequestered membrane. (**C**) A lysosome with an enclosed organelle in the process of degradation. (**D**) An autolysosome clearly enclosing a damaged organelle (in this case, a mitochondrion).

To further confirm the completion of autophagy, we examined kidney samples by transmission electron microscopy. The autolysosome, which has a single limiting membrane and contains cytoplasmic/organellar materials at various stages of degradation, can be distinguished from the autophagosome (containing a double limiting membrane) by electron microscopy. We therefore categorized the autophagic structures into 4 groups as follows (Fig. 2A–D): A) Autophagosome with clear double membrane structures, B) Autophagosome being engulfed process or just engulfed by a sequestered membrane, C) Lysosome, possibly autolysosome with enclosed organelles in varying states of degradation, D) Autolysosome clearly enclosing recognizeable specific organelles or organelle fragments. For analytic purposes, we treated both A) and B) as autophagosomes, and both C) and D) as autolysosomes.

**Figure 3:**
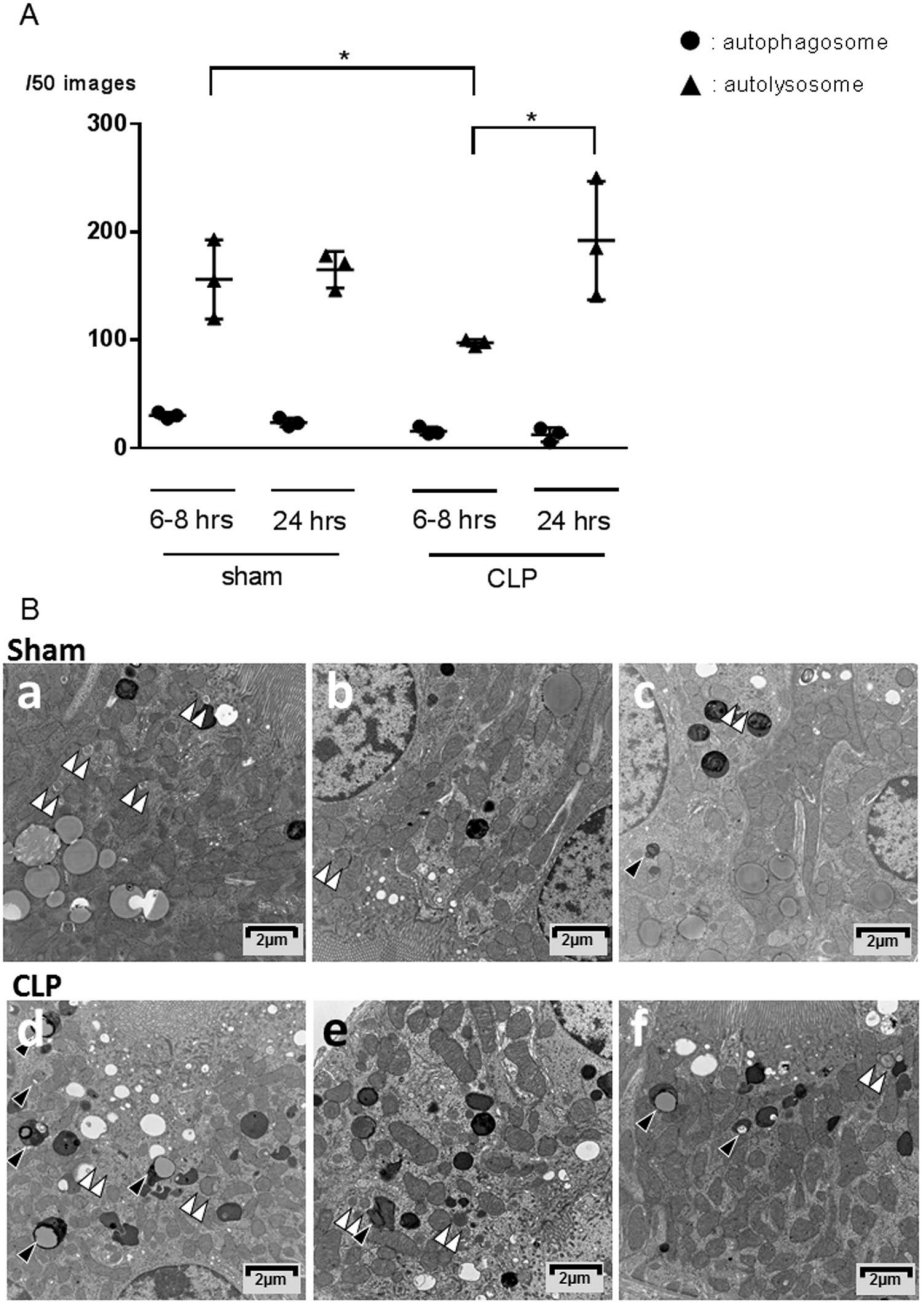
Electron microscopic analysis of the proximal tubules. (**A**) The number of autophagosomes and autolysosomes in CLP and sham animals are compared. All data were expressed as the median ± interquartile range. Data were analyzed for statistical significance using the Mann-Whitney test (*P < 0.05; n = 3 in each group). (**B**) Images of electron microscopy of the proximal convoluted tubule of kidney at 24 hrs after operation; a–c: Kidney sample obtained from sham-operated mice. Organelles in the proximal convoluted tubule cells are generally intact; d–e: CLP-operated mice. Double arrow heads identify complex structures bounded by two membranes (autophagosomes); arrow heads identify single membrane-bound lysosomal complexes with degraded organellar content (autolysosomes).

The number of autophagosomes and autolysosomes in proximal tubular cells per 50 images for each mouse from sham and CLP mice were counted at 6–8 hrs and 24 hrs after operation (Fig. 3A). In CLP mice, the number of autolysosomes at 24 hrs after operation significantly increased compared to that of 6–8 hrs (97.33 ± 1.76: 192.0 ± 31.7, P < 0.05). In sham operated mice, the number of autolysosomes did not increase significantly between 6–8 and 24 hrs after operation (156.0 ± 21.1: 165.0 ± 9.79, P = 0.72). This indicates that autophagy kinetics in renal proximal tubule epithelial cells are accelerated early in sepsis. In CLP mice, the number of autolysosomes at 24 hrs after operation was slightly increased compared to that of sham mice (192.0 ± 54.8: 165.0 ± 16.8, P = 0.70). In contrast, the number of proximal tubular epithelial autophagosomes in CLP mice decreased relative to that in sham at 24 hrs after CLP (12.33 ± 6.66: 23.67 ± 4.04, P = 0.10) (Fig. 3A). This result supports immunofluorescent data that indicate that autophagy flux is already in a trend of stagnation by that time. As shown in Fig. 3B, autolysosomes with enclosed degraded organelles are prominent in CLP kidney proximal tubules.

### Stagnation of autophagy flux in the kidney of murine septic model

**Figure 4:**
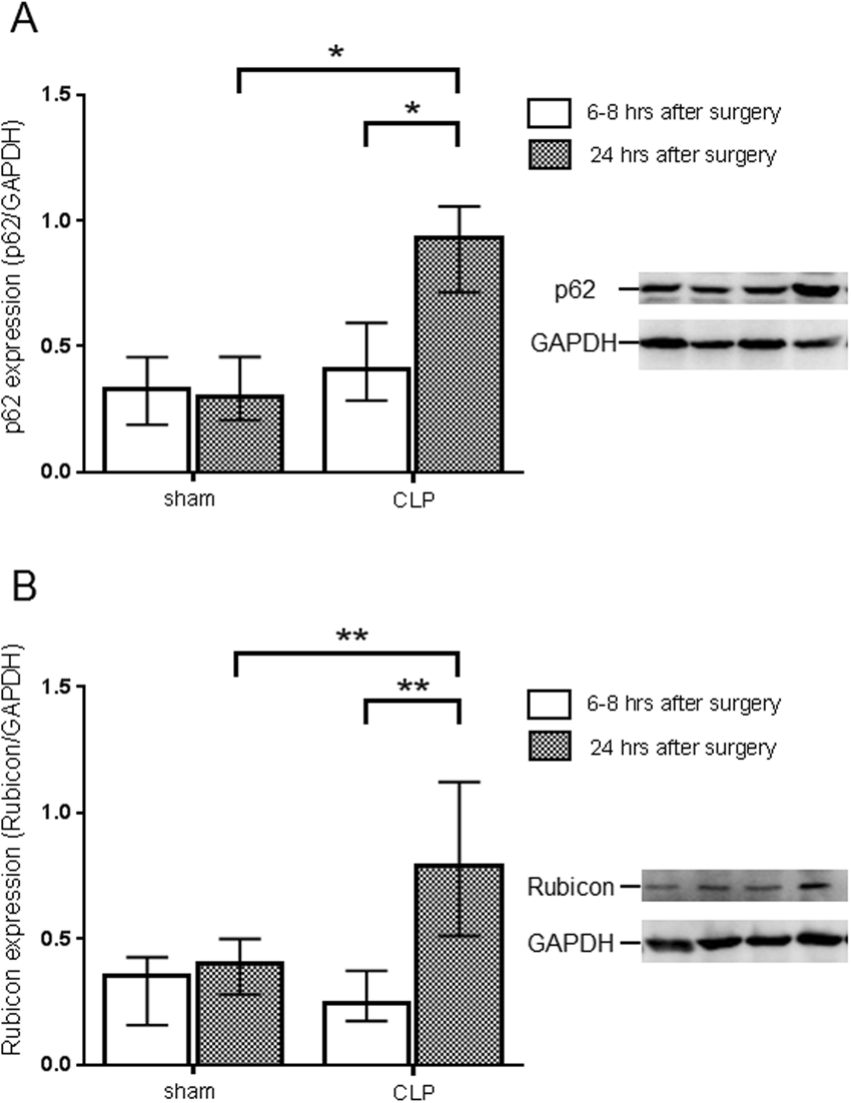
Evaluation of autophagy flux change in the kidney of cecal ligation puncture (CLP). Relative expression of p62 protein (**A**) and Rubicon (**B**) in the kidney at 6–8 and 24 hrs after sham or CLP operation in C57BL/6 N mice. The amount of p62 protein and Rubicon was normalized to that of GAPDH by evaluation of band intensity from western blotting. All data were expressed as the median ± interquartile range. Data were analyzed for statistical significance using the Mann-Whitney test (*P < 0.05, **P < 0.01; n = 4–6 in each group). Whole image of the blot is shown in supplementary information.

Since the number of autolysosomes increased in the kidney in early sepsis, we examined autophagy flux biochemically by measuring the amount of p62 and Rubicon. p62 protein accumulates when autophagy is inhibited^33^. Rubicon is an inhibitor protein that blocks the fusion of an autophagosome to a lysosome^34^. There was a significant increase in the amount of p62 protein at 24 hrs after CLP treatment compared to that at 6–8 hrs in the kidney in C57BL/6 N mice (Fig. 4A). In contrast, the amount of p62 did not change in the 24 hr period after treatment in sham operated mice. The expression of Rubicon protein in the kidney also increased significantly at 24 hrs after CLP treatment but not in shams when compared to values obtained at 6–8 hrs (Fig. 4B). These results suggest suppression of autophagy flux in the septic kidney.

### Protective role of autophagy in the kidney of the septic model mice

**Figure 5:**
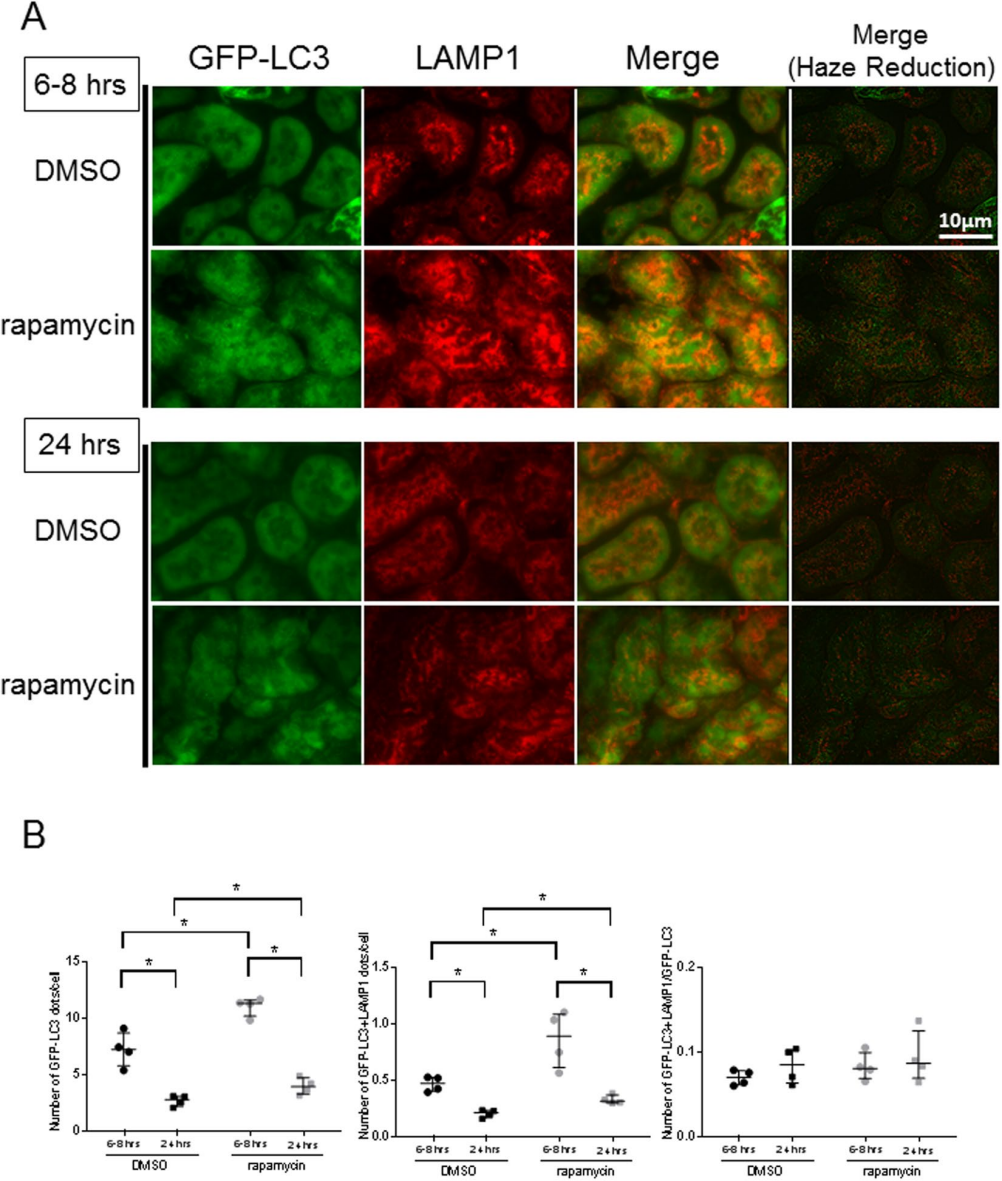
Acceleration of autophagy process by rapamycin. Green fluorescent protein- microtubule-associated protein light chain 3 (GFP-LC3) transgenic mice were administered saline or rapamycin (10 mg/kg intraperitoneally) at 1 hr after cecal ligation and puncture (CLP) surgery. Samples were obtained from 6–8 or 24 hrs after surgery. (**A**) Confocal images of kidney samples obtained from GFP-LC3 transgenic mice with or without rapamycin treatment (400× ). (**B**) The number of GFP-LC3 or GFP-LC3 + lysosome-associated membrane protein type 1 (LAMP1) dots per cellular confocal image obtained from GFP-LC3 transgenic mice with or without rapamycin treatment was quantified at 6–8 and 24 hrs after CLP. We counted the number of dots within renal tubular cells; dots within glomerular cells were not counted. The ratio of GFP-LC3 and GFP-LC3 + LAMP1 dots was calculated. The ratio of GFP-LC3 and GFP-LC3 + LAMP1 dots was calculated. All data are expressed as the median ± interquartile range. Data were analyzed for statistical significance using the Mann-Whitney test. (*P < 0.05; n = 50 cells /animal; n = 4 animals).

**Figure 6:**
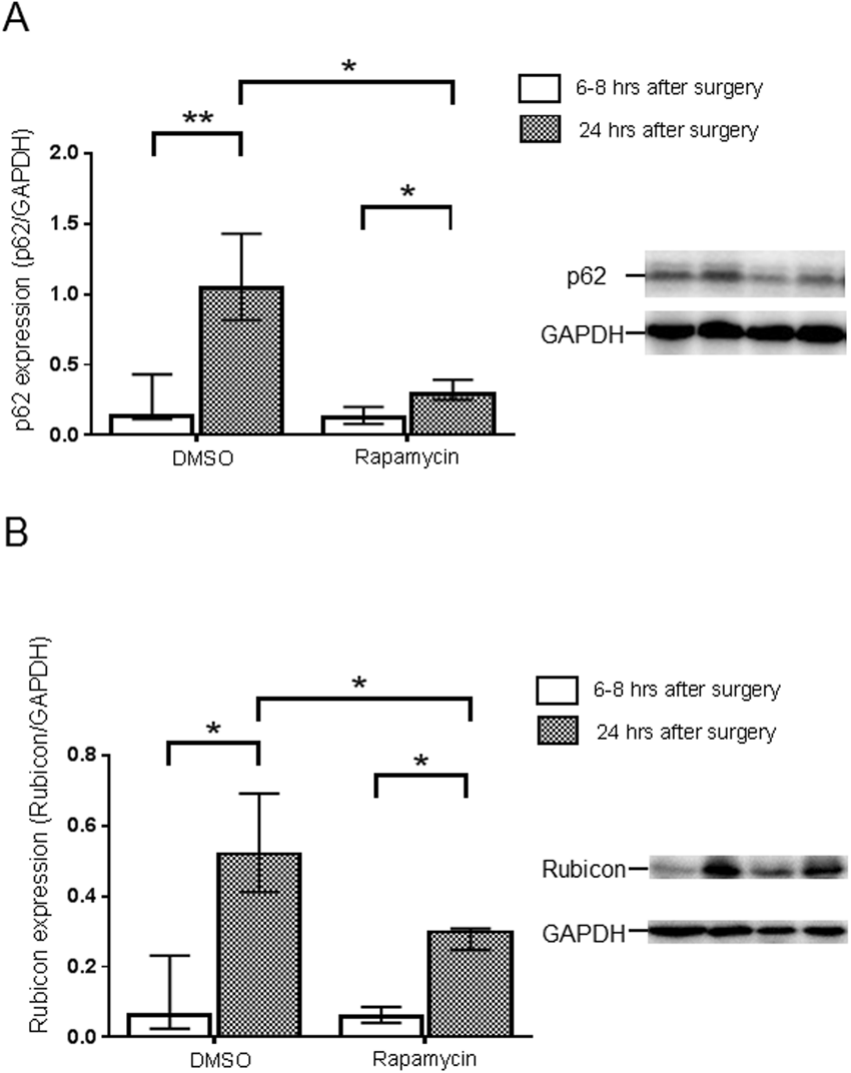
Rapamycin improves autophagy flux in cecal ligation and puncture (CLP) mice. Relative expression of p62 protein and Rubicon in the kidney with or without rapamycin treatment at 6–8 and 24 hrs after CLP operation. The amount of p62 protein and Rubicon was normalized to that of GAPDH by evaluation of band intensity from western blotting. All data were expressed as the mean ± SD. Data were analyzed for statistical significance using the Mann-Whitney test (*P < 0.05, **P < 0.01; n = 4–6 in each group). Whole image of the blot is shown in supplementary information.

**Figure 7:**
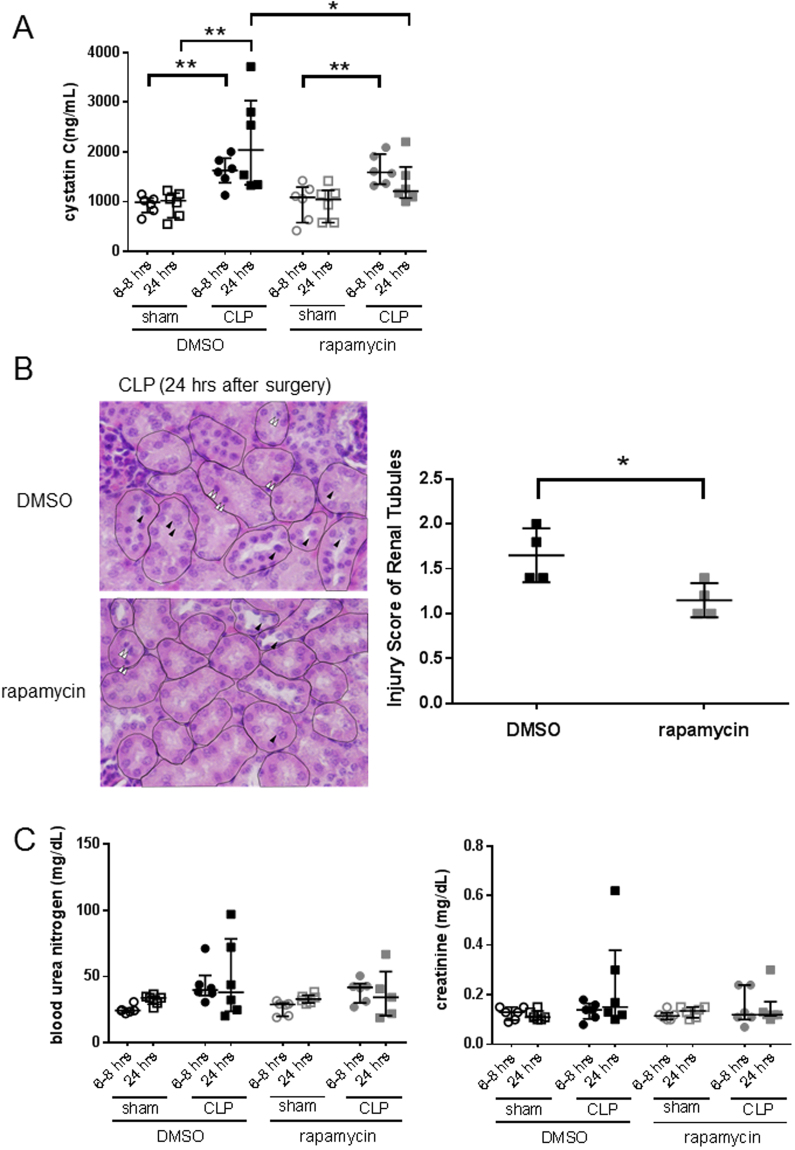
Promotion of autophagy attenuates cecal ligation and puncture (CLP)-induced kidney injury. Nephropathy as defined by serum (**A**) cystatin C, (**C**) blood urea nitrogen and creatinine levels. Samples were obtained from either sham-operated or CLP mice with or without rapamycin treatment 6–8 and 24 hrs after surgery. All data were expressed as the median ± interquartile range. Data were analyzed for statistical significance using the Mann-Whitney test (*P < 0.05, **P < 0.01, n = 6 in each group). (**B**) Pathological examination. Histological findings of mouse kidney by hematoxylin and eosin staining (100×). Kidney tissue was obtained from CLP mice with rapamycin or DMSO treatment at 24 hrs after surgery. Black arrow heads show parts of cells shedding, and white double arrow heads show chromatin condensation. Renal tubules are surrounded by dotted circular lines. CLP mice kidneys (24 hrs after surgery) treated by rapamycin had significantly lower kidney injury scores than those of treated by DMSO. All data were expressed as the median ± interquartile range. Data were analyzed for statistical significance using the unpaired t test (*P < 0.05; n = 4 animals).

The autophagy machinery in the kidney of septic animals was activated after CLP, but autophagy flux diminished over time. To determine whether these changes were beneficial or detrimental to the septic animal, we assessed the effect of rapamycin on selected indices of autophagy, as well as traditional measures of renal function. Rapamycin promotes autophagy by inhibiting mammalian target of rapamycin (mTOR)^35^. First, we confirmed the efficacy of rapamycin using the GFP-LC3 transgenic mice in the kidney of CLP model (Fig. 5A). As expected, the number of autophagic structures significantly increased at both 6–8hrs and 24hrs post-CLP after infusion of rapamycin (Fig. 5B). Furthermore, in kidneys of rapamycin treated CLP mice, both p62 and Rubicon were reduced significantly at 24 hrs compared to kidneys from non-treated CLP mice (Fig. 6A and B). This result shows that rapamycin prevents stagnation of autophagy flux in the kidney of CLP mice. Cystatin C is a sensitive marker for the early phase of renal dysfunction. Serum cystatin C concentration was significantly higher in CLP operated groups both at 6–8 and 24 hrs when compared to shams (indicative of renal dysfunction), whereas cystatin C concentration was significantly lower at 24 hrs after surgery in the rapamycin treated group compared to CLP controls (Fig. 7A). Furthermore, histological analysis revealed that CLP mice treated by rapamycin had significantly lower renal damage than those treated by DMSO 24 hrs after surgery (Fig. 7B). On the other hand, there were no significant differences in the serum concentration of BUN and creatinine between controls and the rapamycin-treated group at all time points assessed (Fig. 7C). These results suggest that accelerating autophagy protects animals from renal damage in the early phase of sepsis.

## Discussion

In this report, we analyzed autophagic structures in renal proximal tubular epithelial cells in septic mice using electron microscopy. The number of autolysosomes in the kidney of CLP treated mice increased within 24 hrs after operation. However, biochemical analysis revealed that autophagy flux was suppressed with progression of sepsis after 6–8 hrs. Impaired renal function in the 24-hr CLP model was mitigated by enhancement of autophagy with rapamycin. These results raise the possibility that stimulation of autophagy might improve renal function in sepsis.

Sepsis is a major cause of acute kidney injury (AKI), accounting for nearly half of all AKI cases in critical care medicine^36^. Some patients with septic AKI experience complete recovery of renal function and improvement in their general condition. However, others develop irreversible renal dysfunction. Autophagosomes are significantly increased in kidneys from autopsied patients with sepsis^37^, suggesting that regulation of autophagy may contribute to the recovery of renal function in patients with septic AKI. This assumption is supported by a recent study showing enhancement of autophagy to ameliorate septic AKI in experimental animals^38^.

Studies have also shown that the organ-protective effects of autophagy in AKI are triggered by factors other than sepsis. In proximal tubule-specific Atg 5 knockout mice with cisplatin-induced kidney injury (DNA damage was increased by cisplatin), autophagy exerts a protective effect against DNA damage and suppresses the development of renal dysfunction^39^. Autophagosomes are abundant in the damaged proximal tubular renal cells in a murine renal ischemia reperfusion model^40^ and in Atg 5 knockout mice with suppressed autophagy, apoptosis and tubular cell damage are more marked after reperfusion injury when compared to wild-type mice. In this context, apoptosis is thought to be induced by reactive oxygen species (ROS) production in response to mitochondrial dysfunction, suggesting that autophagy protects renal tubular cells by removing damaged mitochondria^32^. Of course, ROS is not the sole cause of tubular cell damage associated with damaged mitochondria. It has also been suggested that acidosis stimulates glutamine uptake into the proximal tubule and generates more bicarbonate and ammonia in order to neutralize the acidity, which increases the burden on mitochondria. In order to remove the mitochondria damaged as a result, autophagy is activated. When autophagy fails (or is down-regulated by other means), accumulation of damaged mitochondria leads to tubular dysfunction^38^.

Recently, attention has focused on an autophagy regulatory protein known as Rubicon. This protein forms a complex with Beclin 1 and inhibits fusion of autophagosomes and autolysosomes^34^. On the other hand, Rubicon participates in non-canonical autophagy (e.g. LC3-associated phagocytosis [LAP]). Notably, Rubicon is increased after induction of sepsis and continues to increase between 6–8 and 24 hrs after CLP. These results suggests that autophagy is suppressed by sepsis itself in the septic kidney. The reason that Rubicon increases with time in CLP may be the result of inflammation which promoted antigen uptake by LAP. Increase in Rubicon brings about stagnation of autophagy which may result in organ dysfunction due to accumulation of damaged organelle. Interestingly, p62 accumulation promotes caspase-induced apoptosis^41^, and this mechanism could contribute to renal impairment through proximal tubular cell apoptosis during sepsis. The latter observation emphasizes that crosstalk between autophagy and apoptosis might play an important role in the pathophysiology of septic AKI.

Autophagic structures in renal proximal tubules were evaluated by electron microscopic analysis using consensus definitions for both autophagosomes and autolysosomes. The autolysosome counts in the experimental mice at 6–8 hrs after CLP is significantly lower than those after sham procedure. This observation suggest that autophagy is stagnated as early as 6–8 hrs in the proximal tubules in septic mice, although the number of autophagic structures increased in early phase and decreased thereafter in entire renal tubules as assessed by immunofluorescent microscopy. Throughout the time course of our studies, the fraction of GFP-LC3 + LAMP1 dots/GFP-LC3 dots did not change (Fig. 1C), which means the speed of autophagy process did not increase in CLP. Also, the ratio of ultrastructurally-observed autolysosomes to autophagosomes at 24 hrs after CLP dramatically increased (Fig. 3A), providing morphologic evidence of autophagy flux stagnation in CLP.

The dynamics of autophagy differ in the kidney (based on the current study) from those in the liver in the CLP sepsis mouse model. This is not an unexpected result, considering that regulation of autophagy appears to be organ specific^42^. It is possible that initial activation of the autophagy machinery is delayed in the kidney of the septic mice compared to sham mice. Renal tubular cells may sense environmental change, such as blood flow and pH caused by sepsis, and initially reserve activation of autophagy machinery. This might account for why the number of autophagosomes and autolysosomes in the sham mice was higher than those of CLP mice early in sepsis. Further study is required to elucidate the mechanism and factors that activate or inhibit the autophagic machinery in sepsis.

Rapamycin, which induces autophagy, may have reduced the extent of AKI in sepsis. In particular, while the production of autophagosomes decreases over time in the kidneys of CLP mice, rapamycin seems to have alleviated this stagnation of autophagy flux by increasing autophagosome production. In addition, serum cystatin C level decreased in rapamycin treated animals compared with untreated controls at 24 h after CLP (p = 0.04). Finally, histological analysis revealed that the proportion of injured tubule decreased in the rapamycin treated group compared with untreated controls at 24hrs after CLP (p = 0.03). This result indicates that autophagy plays a protective role in sepsis induced kidney injury. Interestingly, there were no significant differences in blood concentrations of BUN and creatinine between the two groups. We speculate that CLP mice did not have enough kidney damage to effect significant alterations in BUN and Creatinine within the study time horizon of 24 hours. On the other hand, cystatin C reflects the degree of kidney injury early and sensitively and significant differences between treated and untreated populations were observed. Paradoxically, rapamycin is known to induce renal dysfunction by suppressing mTOR, and amTOR knockout mice reportedly develop proteinuria and glomerular sclerosis as they age^43^. It is notable, therefore, that in the present study, renal function improved despite the administration of rapamycin, suggesting that enhancement of autophagy has a more immediate effect on renal function that mTOR suppression, resulting in mitigation of progressive renal failure. Because endotoxemia is associated with activation of mTORC1 (mTOR complex 1), it is likely that mTORC1 inhibition enhances autophagy flux^29^. Importantly, mTORC1 is also associated with other vital phenomena (including cell growth, metabolism, fission, protein synthesis and gene transcription), suggesting that rapamycin likely exerts its influence on pathways other than autophagy. While increased autophagy is a possible reason for the improved pathology and cystatin C values, it is not proven by the present study. Hence, further study is needed by using a models built on kidney specific transgenic animals.

Previous reports^44,45^ have shown that kidney function can be improved by promoting autophagy of the sepsis model. Our report embellishes this understanding by documenting changes in the number of autolysosomes as well as autophagosomes, using immunofluorescent microscopy and electron microscopy. Furthermore, we have clarified that Rubicon is involved in the control of autophagy and organ damage in septic AKI. On the other hand, direct link between increased autophagy and changes of the biochemical data such as cystatin C and Rubicon were not proven in the present study. Development of organ specific autophagy inducible mice models will overcome these limitations. In the future, in order to clinically apply the organ protective effect of autophagy in sepsis, it is important to know in which stage of the autophagy cascade to intervene. It is important to acknowledge that the roles of autophagy and other cell death pathways in sepsis-associated organ dysfunction are effectively dose-dependent, varying with the severity of sepsis^46^. Further studies are needed to develop an effective treatment strategy for septic AKI (drugs and optimal timing of administration) with this in mind. It is also important to clarify the patient population that benefits most from autophagy stimulation.

## Conclusion

Autophagy flux diminishes in the kidney of septic mice over time. Acceleration of autophagy improves renal function, as monitored by serum cystatin C. These results suggest that autophagy plays a protective role against sepsis-induced kidney injury and may mitigate other physiological abnormalities during sepsis as well. Importantly, the appropriate timing of autophagy upregulation in the clinical setting has not yet been determined, since no method of *in vivo* monitoring of autophagy in humans has yet been established. In addition, because autophagy regulation is organ specific, additional analysis of the mechanisms underlying autophagy in different organs during sepsis is required.

## Electronic supplementary material

Supplementary Information
