# Supplementary material for: Influence of autophagy on acute kidney injury in a murine cecal ligation and puncture sepsis model
Source: Sci Rep. 2018 Jan 18;8:1050. doi: 10.1038/s41598-018-19350-w (PMC5773584; doi:10.1038/s41598-018-19350-w)
Supplement: Supplementary file 1 — Supplementary Information [file 41598_2018_19350_MOESM1_ESM.pdf]

**Title:**

# Influence of autophagy on acute kidney injury in a murine cecal ligation and puncture sepsis model

**Satoshi Sunahara, MD* , Eizo Watanabe, MD, PhD* , Masahiko Hatano, MD, PhD†‡, Paul E Swanson, PhD¶, Takehiko Oami, MD, PhD* , Lisa Fujimura, PhD†, Youichi Teratake, PhD†, Takashi Shimazui*, Chiwei Lee, MD, PhD §, Shigeto Oda, MD, PhD***

***Department of Emergency and Critical Care Medicine, Graduate School of Medicine, Chiba University**
**†Biomedical Research Center, Chiba University**
**‡Department of Biomedical Science, Chiba University Graduate School of Medicine**
**¶Department of Pathology, University of Washington School of Medicine**
**§ Department of Nephrology, Chiba University Graduate School of Medicine**

Corresponding Author: Eizo Watanabe, MD, PhD
1-8-1 Inohana, Chuo-ku, Chiba City, Chiba 260-8670, Japan
Department of Emergency and Critical Care Medicine, Graduate School of Medicine, Chiba University
E-mail: watanabee@faculty.chiba-u.jp
Phone: +81-43-226-2372
Supported by Grants-in-Aid for Scientific Research from the Ministry of Education, Culture, Sports Science and Technology, Japan and by Grant for Young Doctors of Abdominal Emergency Clinical Resarch from the Japanese Society for Abdominal Emergency Medicine.

【LC3】

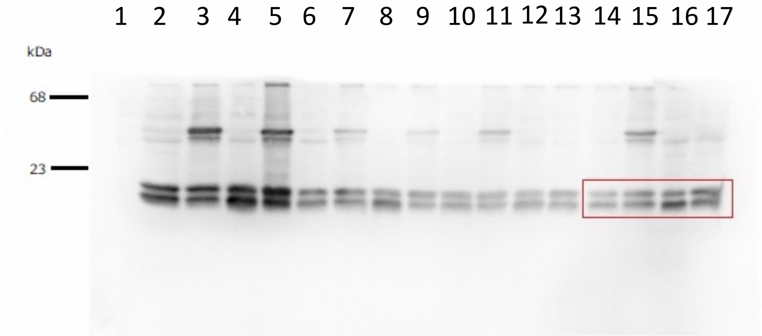


【GAPDH】

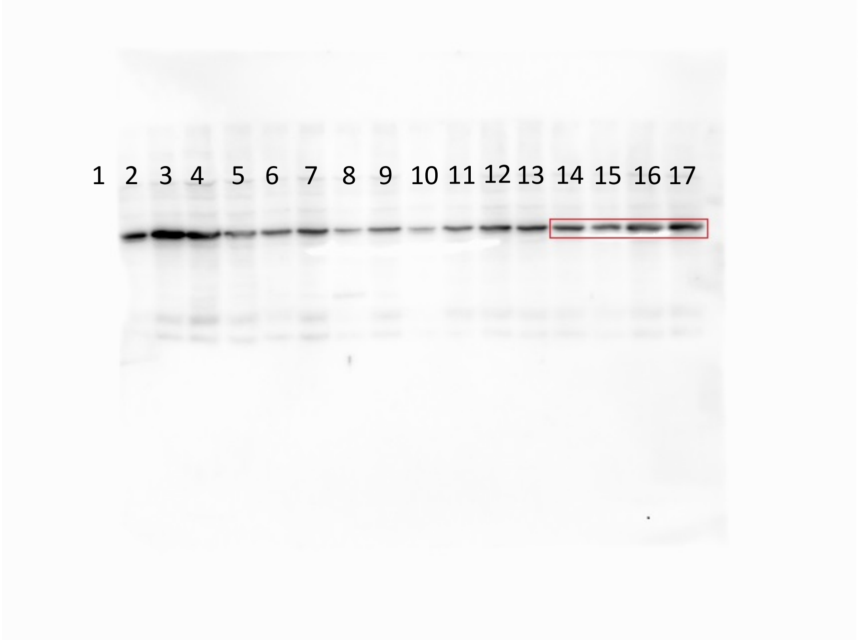


**Figure S1** Whole images of Western blotting in Fig.1A.

Upper figure indicates LC3 expression and lower figure indicates GAPDH as a loading control. Lanes 14 , 15, 16, and 17 were cropped and shown as a representative figure in the main text. Lane1: Molecular weight marker. Since the marker was transferred very faintly, we marked the position of each molecular weight with reference to the position of the marker on each gel. Lane 14: sham, 6-8 hrs; lane 15: sham, 24 hrs; lane 16: CLP, 6-8 hrs; lane 17: CLP, 24hrs.

The samples loaded on other lanes are test samples which are not related to this experiment. These samples were loaded to fill all the wells for straight electrophoresis.

【p62】

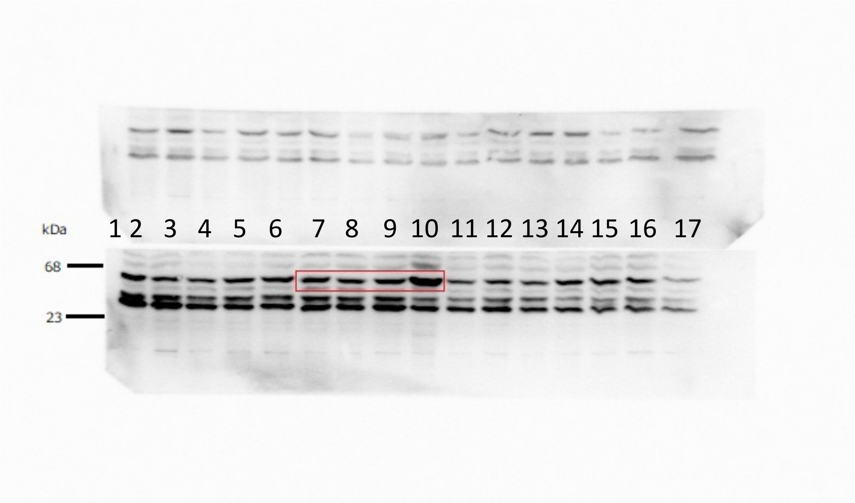


【GAPDH】

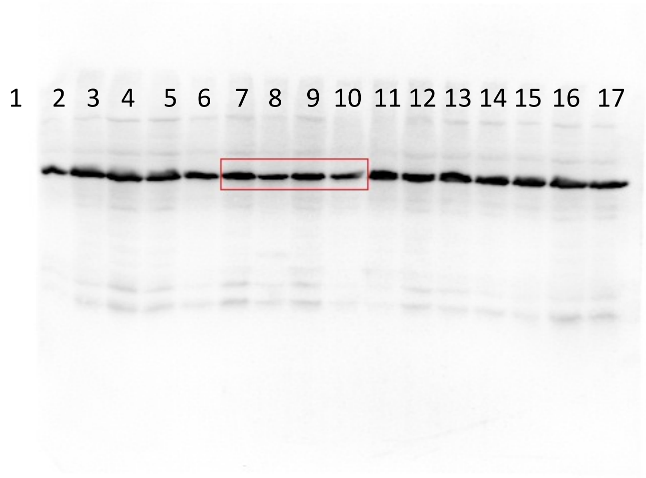


**Figure S2A** Whole images of Western blotting in Fig.4A.
Upper figure indicates p62 expression and lower figure indicates GAPDH as a loading control. Lanes 7 , 8, 9, and 10 were cropped and shown as a representative figure in the main text. Lane1: Molecular weight marker. Since the marker was transferred very faintly, we marked the position of each molecular weight with reference to the position of the marker on each gel. Lane 7: sham, 6-8 hrs; lane 8: sham, 24 hrs; lane 9: CLP, 6-8 hrs; lane 10: CLP, 24hrs.
The samples loaded on other lanes are test samples which are not related to this experiment. These samples were loaded to fill all the wells for straight electrophoresis.

【Rubicon】

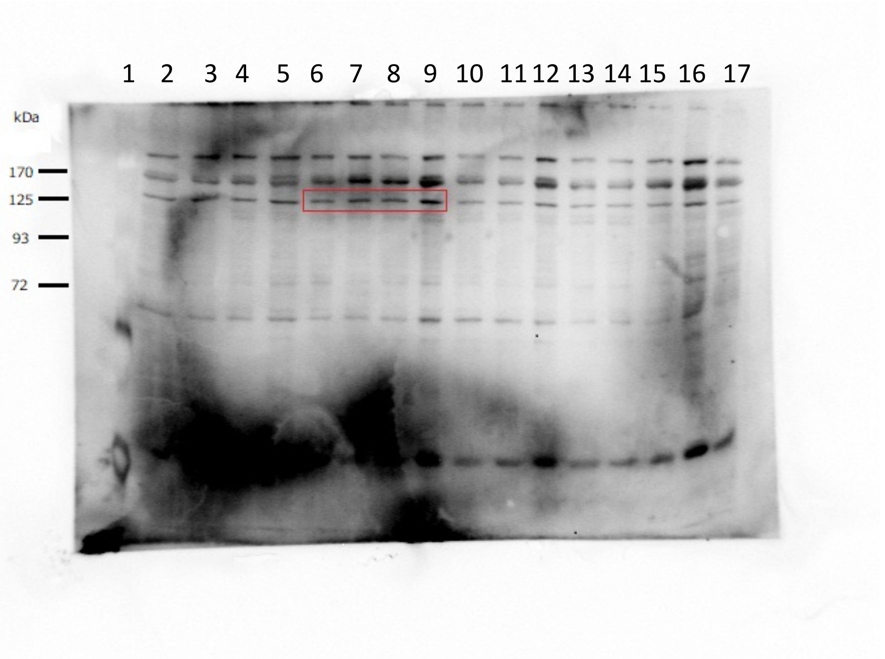


【GAPDH】

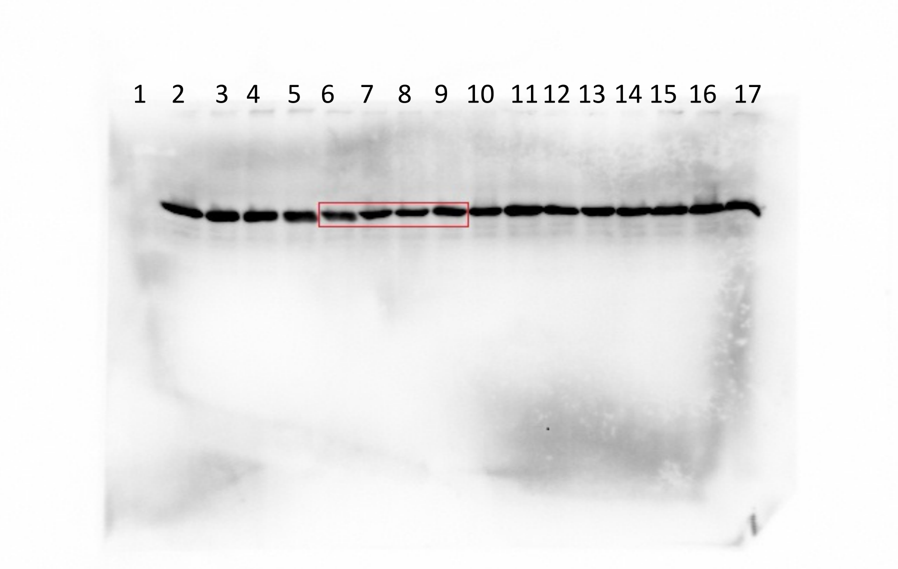


**Figure S2B** Whole images of Western blotting in Fig.4B.
Upper figure indicates Rubicon expression and lower figure indicates GAPDH as a loading control. Lanes 6 , 7, 8, and 9 were cropped and shown as a representative figure in the main text. Lane1: Molecular weight marker. Since the marker was transferred very faintly, we marked the position of each molecular weight with reference to the position of the marker on each gel. Lane 6: sham, 6-8 hrs; lane 7: sham, 24 hrs; lane 8: CLP, 6-8 hrs; lane 9:CLP, 24hrs.
The samples loaded on other lanes are test samples which are not related to this experiment. These samples were loaded to fill all the wells for straight electrophoresis.

【p62】

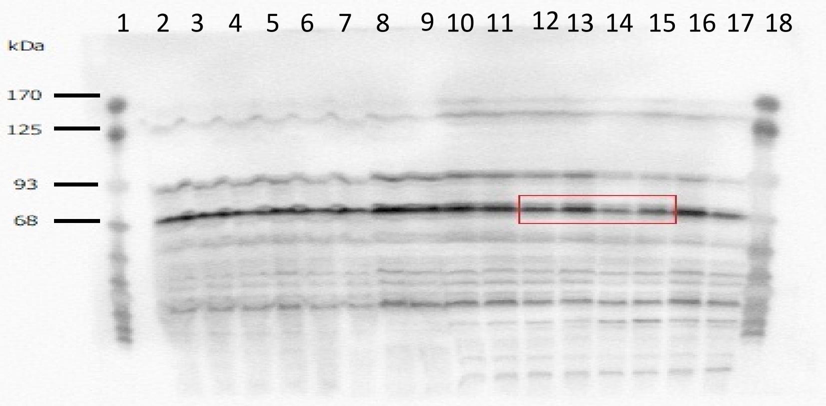


【GAPDH】

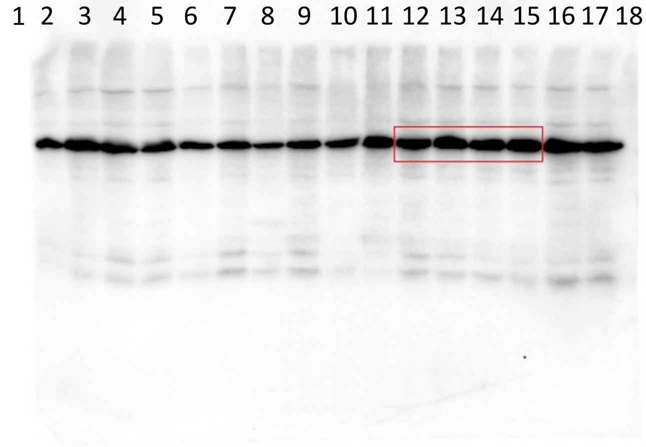


**Figure S3A** Whole images of Western blotting in Fig.6A.

Upper figure indicates p62 expression and lower figure indicates GAPDH as a loading control. Lanes 12 , 13, 14, and 15 were cropped and shown as a representative figure in the main text. Lane1 and 18: Molecular weight marker. Since the marker was transferred very faintly, we marked the position of each molecular weight with reference to the position of the marker on each gel. Lane 12: CLP administrated DMSO, 6-8 hrs; lane 13: CLP administrated DMSO, 24 hrs; lane 14: CLP administrated rapamycin, 6-8 hrs; lane 15: CLP administrated rapamycin, 24 hrs.

The samples loaded on other lanes are test samples which are not related to this experiment. These samples were loaded to fill all the wells for straight electrophoresis.

【Rubicon】

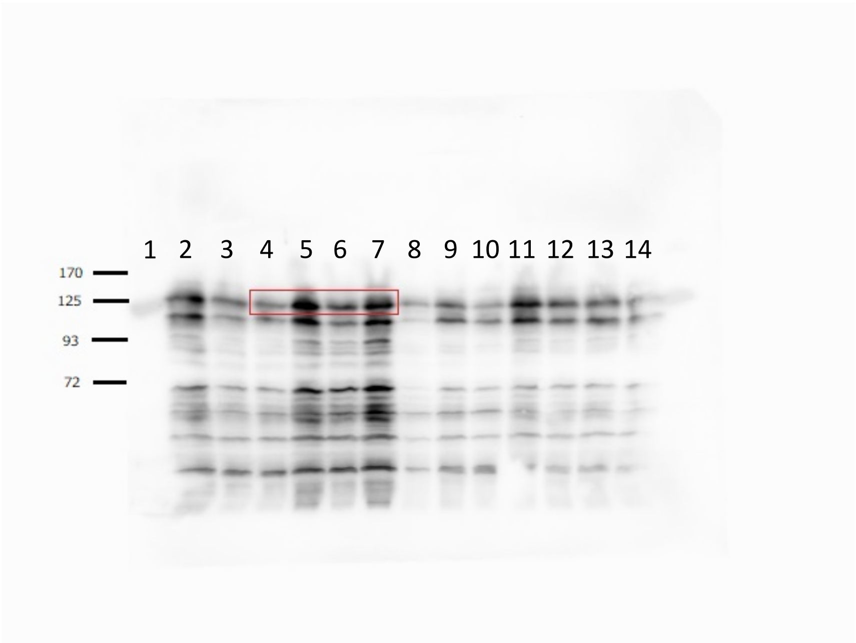


【GAPDH】

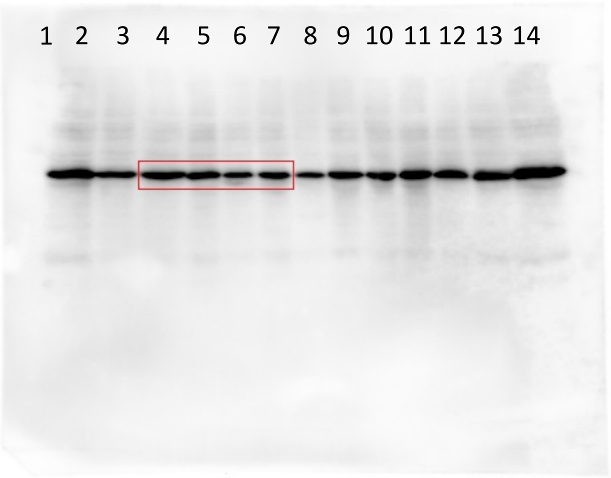


**Figure S3B** Whole images of Western blotting in Fig.6B.

Upper figure indicates Rubicon expression and lower figure indicates GAPDH as a loading control. Lanes 4 , 5, 6, and 7 were cropped and shown as a representative figure in the main text. Lane1: Molecular weight marker. Since the marker was transferred very faintly, we marked the position of each molecular weight with reference to the position of the marker on each gel. Lane 4: CLP administrated DMSO, 6-8 hrs; lane 5: CLP administrated DMSO, 24 hrs; lane 6: CLP administrated rapamycin, 6-8 hrs; lane 7: CLP administrated rapamycin, 24 hrs.

The samples loaded on other lanes are test samples which are not related to this experiment. These samples were loaded to fill all the wells for straight electrophoresis.
